# The Alcoholic Problem*U. S. Senate Document 48. “Some Scientific Conclusions Concerning the Alcoholic Problem and Its Practical Relations to Life.” Papers read at the semi-annual meeting of the American Society for the Study of Alcohol and Other Drug Narcotics, at Washington, D. C., March 17, 18, 19, 1909. Government Printing Office, Washington, 1909. Presented by Senator Gallinger, May 17, 1909. Ordered to be printed.

**Published:** 1910-02

**Authors:** 


### EDITORIAL DEPARTMENT.

### THE ALCOHOLIC PROBLEM.*

The most hopeful sign of the times is the fact that the United States government is awakening to the importance—economically and otherwise—of dealing with the most destructive evil of our civilization, the indiscriminate sale and use of that most deadly narcotic poison—alcohol. For the first time has Congress consented to listen to reason on the subject, to earnestly heed the voice of science in its showing that alcohol strikes at the fundamental interest of society and is ruinous to the public health and morals—sapping the foundations of our social fabric. The papers read at the March meeting of the American Society for the Study of Alcohol were submitted to Congress, and the Senate was so impressed with them and the deductions therefrom that they were ordered printed and distributed at government expense. This leads to the hope that soon effective legislation will be enacted that will materially lessen if it does not remove the evil of drunkenness. These papers were submitted by some of the ablest and most distinguished biologists, pathologists and psychiatrists in America—men who for years have made a deep laboratory study of the effects of alcohol or man. Amongst the contributors are Kellogg, Hughes, Berkeley, Crothers, W. S. Hall, Howard Kelley (Johns Hopkins), Marcy and numerous others—acknowledged authorities. The publication, "Document 48, United States Senate," is one of the greatest interest and importance, and every physician who feels any concern for his country or who has any sympathy for the poor, deluded, ignorant people who poison themselves with the sanction of the law should read it and especially study the conclusions of these scientific men. A copy can be had free on request from any of the Senators. The "Conclusions" may be briefly summarized as follows:

Alcohol is not a stimulant or tonic, but a powerful narcotic poison. It is not a food, in any sense, but an adventitious or for-

*U. S. Senate Document 48. "Some Scientific Conclusions Concerning the Alcoholic Problem and Its Practical Relations to Life." Papers read at the semi-annual meeting of the American Society for the Study of Alcohol and Other Drug Narcotics, at Washington, D. C., March 17, 18, 19, 1909. Government Printing Office, Washington, 1909. Presented by Senator Gallinger, May 17, 1909. Ordered to be printed.

eign substance which the system makes every effort to eliminate. It is not a useful drug, and is not necessary in the treatment of disease. It paralyzes the respiratory and cardiac nerve centers; it paralyzes (puts to sleep) the phagocytes—those vigilant, wakeful, active and intelligent little fellows—the "police force" of the system whose duty it is to resist and destroy invading disease germs, and hence weakens one's powers of resistance, and predisposes to disease; it shortens life. It leads to insanity, and is the cause of insanity and crime in a very large proportion of cases. And as such I may safely say that it is the *most potent factor in the swift decay of the human race.* To license the sale of such an agent is nothing short of *idiocy!* Send for the book and read it, and join us in the hope that Congress will cease to authorize the Toms, Dicks and Harrys to make criminals, paupers, lunatics and widows and orphans of our people.

